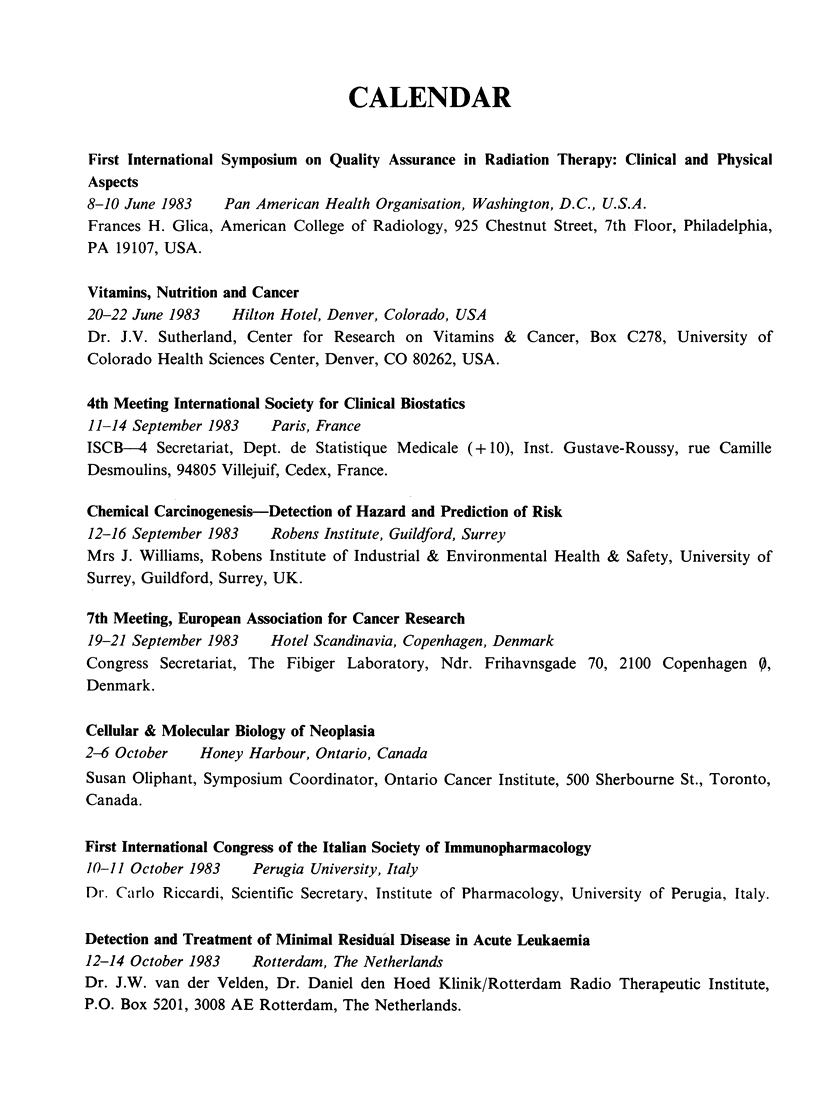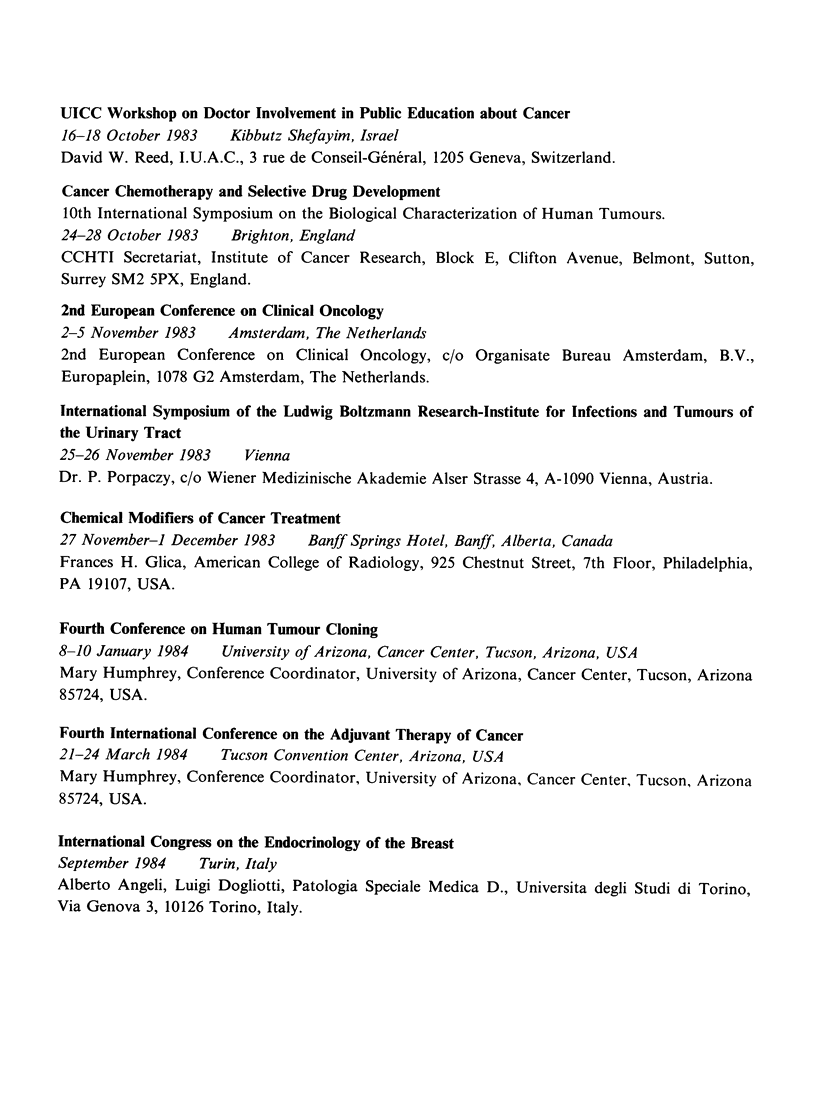# Calendar

**Published:** 1983-05

**Authors:** 


### CALENDAR

**First International Symposium on Quality Assurance in Radiation Therapy: Clinical and Physical Aspects**
*8–10 June 1983 Pan American Health Organisation, Washington, D.C., U.S.A.*
Frances H. Glica, American College of Radiology, 925 Chestnut Street, 7th Floor, Philadelphia, PA 19107, USA.

**Vitamins, Nutrition and Cancer**
*20–22 June 1983 Hilton Hotel, Denver, Colorado, USA*
Dr. J.V. Sutherland, Center for Research on Vitamins & Cancer, Box C278, University of Colorado Health Sciences Center, Denver, CO 80262, USA.

**4th Meeting International Society for Clinical Biostatics**
*11–14 September 1983 Paris, France*
ISCB—4 Secretariat, Dept. de Statistique Medicale (+10), Inst. Gustave-Roussy, rue Camille Desmoulins, 94805 Villejuif, Cedex, France.

**Chemical Carcinogenesis—Detection of Hazard and Prediction of Risk**
*12–16 September 1983 Robens Institute, Guildford, Surrey*
Mrs J. Williams, Robens Institute of Industrial & Environmental Health & Safety, University of Surrey, Guildford, Surrey, UK.

**7th Meeting, European Association for Cancer Research**
*19–21 September 1983 Hotel Scandinavia, Copenhagen, Denmark*
Congress Secretariat, The Fibiger Laboratory, Ndr. Frihavnsgade 70, 2100 Copenhagen Ø, Denmark.

**Cellular & Molecular Biology of Neoplasia**
*2–6 October Honey Harbour, Ontario, Canada*
Susan Oliphant, Symposium Coordinator, Ontario Cancer Institute, 500 Sherbourne St., Toronto, Canada.

**First International Congress of the Italian Society of Immunopharmacology**
*10–11 October 1983 Perugia University, Italy*
Dr. Carlo Riccardi, Scientific Secretary, Institute of Pharmacology, University of Perugia, Italy.

**Detection and Treatment of Minimal Residual Disease in Acute Leukaemia**
*12–14 October 1983 Rotterdam, The Netherlands*
Dr. J.W. van der Velden, Dr. Daniel den Hoed Klinik/Rotterdam Radio Therapeutic Institute, P.O. Box 5201, 3008 AE Rotterdam, The Netherlands.

**UICC Workshop on Doctor Involvement in Public Education about Cancer**
*16–18 October 1983* *Kibbutz Shefayim, Israel*
David W. Reed, I.U.A.C., 3 rue de Conseil-Général, 1205 Geneva, Switzerland.

**Cancer Chemotherapy and Selective Drug Development**
10th International Symposium on the Biological Characterization of Human Tumours.
*24–28 October 1983* *Brighton, England*
CCHTI Secretariat, Institute of Cancer Research, Block E, Clifton Avenue, Belmont, Sutton, Surrey SM2 5PX, England.

**2nd European Conference on Clinical Oncology**
*2–5 November 1983* *Amsterdam, The Netherlands*
2nd European Conference on Clinical Oncology, c/o Organisate Bureau Amsterdam, B.V., Europaplein, 1078 G2 Amsterdam, The Netherlands.

**International Symposium of the Ludwig Boltzmann Research-Institute for Infections and Tumours of the Urinary Tract**
*25–26 November 1983* *Vienna*
Dr. P. Porpaczy, c/o Wiener Medizinische Akademie Alser Strasse 4, A-1090 Vienna, Austria.

**Chemical Modifiers of Cancer Treatment**
*27 November–1 December 1983* *Banff Springs Hotel, Banff, Alberta, Canada*
Frances H. Glica, American College of Radiology, 925 Chestnut Street, 7th Floor, Philadelphia, PA 19107, USA.

**Fourth Conference on Human Tumour Cloning**
*8–10 January 1984* *University of Arizona, Cancer Center, Tucson, Arizona, USA*
Mary Humphrey, Conference Coordinator, University of Arizona, Cancer Center, Tucson, Arizona 85724, USA.

**Fourth International Conference on the Adjuvant Therapy of Cancer**
*21–24 March 1984* *Tucson Convention Center, Arizona, USA*
Mary Humphrey, Conference Coordinator, University of Arizona, Cancer Center, Tucson, Arizona 85724, USA.

**International Congress on the Endocrinology of the Breast**
*September 1984* *Turin, Italy*
Alberto Angeli, Luigi Dogliotti, Patologia Speciale Medica D., Universita degli Studi di Torino, Via Genova 3, 10126 Torino, Italy.